# (*S*)-1-(1-Ferrocenylmethyl-1*H*-benz­imidazol-2-yl)ethanol monohydrate

**DOI:** 10.1107/S1600536809023575

**Published:** 2009-06-27

**Authors:** Rong Xia

**Affiliations:** a Ordered Matter Science Research Center, College of Chemistry and Chemical Engineering, Southeast University, Nanjing 210096, People’s Republic of China

## Abstract

In the structure of the title compound, [Fe(C_5_H_5_)(C_15_H_15_N_2_O)]·H_2_O, the unsubstituted cyclo­penta­diene (Cp) ring is disordered over two positions, with site-occupancy factors of 0.636 (12) and 0.364 (12). The dihedral angles between the planes of the substituted Cp ring and the major and minor components of the disordered ring are 0.8 (3) and 3.4 (6)°, respectively. The crystal packing is stabilized by inter­molecular O—H⋯O hydrogen bonds, forming zigzag chains running parallel to the *a* axis.

## Related literature

For applications of ferrocene compounds, see: Savage *et al.* (2006 ▶); Carr *et al.* (2001 ▶). For the biological and pharmaceutical activity of imidazole and benzimidazole derivatives, see: Matsuno *et al.* (2000 ▶); Garuti *et al.* (1999 ▶). For the synthesis and crystal structure of (±)-1-(1*H*-benzimidazol-2-yl)ethanol, see: Xia & Xu (2008 ▶).
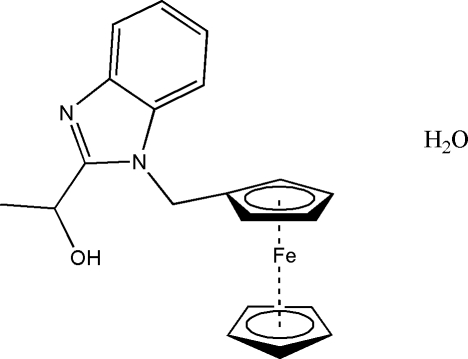


## Experimental

### 

#### Crystal data

[Fe(C_5_H_5_)(C_15_H_15_N_2_O)]·H_2_O
*M* *_r_* = 378.25
Orthorhombic, 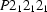

*a* = 7.678 (5) Å
*b* = 12.480 (8) Å
*c* = 19.428 (12) Å
*V* = 1862 (2) Å^3^
*Z* = 4
Mo *K*α radiation
μ = 0.83 mm^−1^
*T* = 293 K
0.40 × 0.35 × 0.30 mm

#### Data collection

Rigaku SCXmini diffractometer
Absorption correction: multi-scan (*CrystalClear*; Rigaku, 2005 ▶) *T*_min_ = 0.724, *T*_max_ = 0.785
19049 measured reflections
4236 independent reflections
3622 reflections with *I* > 2σ(*I*)
*R*_int_ = 0.042

#### Refinement

*R*[*F* ^2^ > 2σ(*F* ^2^)] = 0.043
*wR*(*F* ^2^) = 0.113
*S* = 1.05
4236 reflections
243 parameters
146 restraints
H-atom parameters constrained
Δρ_max_ = 0.32 e Å^−3^
Δρ_min_ = −0.21 e Å^−3^
Absolute structure: Flack (1983 ▶), 1811 Friedel pairs
Flack parameter: 0.03 (2)

### 

Data collection: *CrystalClear* (Rigaku, 2005 ▶); cell refinement: *CrystalClear*; data reduction: *CrystalClear*; program(s) used to solve structure: *SHELXS97* (Sheldrick, 2008 ▶); program(s) used to refine structure: *SHELXL97* (Sheldrick, 2008 ▶); molecular graphics: *SHELXTL* (Sheldrick, 2008 ▶); software used to prepare material for publication: *SHELXTL*.

## Supplementary Material

Crystal structure: contains datablocks I, global. DOI: 10.1107/S1600536809023575/rz2334sup1.cif

Structure factors: contains datablocks I. DOI: 10.1107/S1600536809023575/rz2334Isup2.hkl

Additional supplementary materials:  crystallographic information; 3D view; checkCIF report

## Figures and Tables

**Table 1:** Hydrogen-bond geometry (Å, °)

*D*—H⋯*A*	*D*—H	H⋯*A*	*D*⋯*A*	*D*—H⋯*A*
O*W*—H*WB*⋯O1^i^	0.85	2.37	2.806 (5)	112
O1—H1′′⋯O*W*	0.82	2.34	3.016 (5)	140

Symmetry code: (i) 
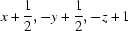
.

**Fig. 1.**
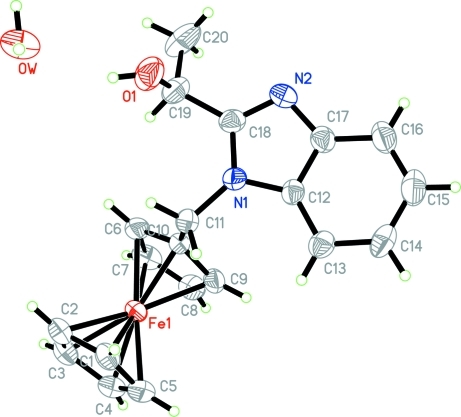
The molecular structure of the title compound with the atomic numbering scheme. Displacement ellipsoids were drawn at the 30% probability level. Only the major component of disorder is shown.

**Fig. 2.**
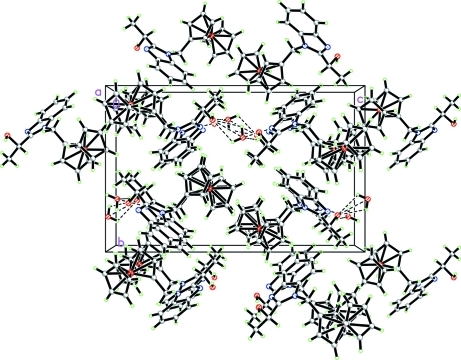
Packing diagram of the title compound viewed along the a axis. Intermolecular O—H···O hydrogen bonds are shown as dashed lines.

## supplementary crystallographic information

### Comment

The organometallic compound ferrocene has found several novel applications due to its stability, spectroscopic properties, electrochemical properties and ease of use (Savage *et al.*, 2006). The ferrocene unit can affect the properties of the binding site and likewise binding event can affect the properties of ferrocene (Carr *et al.*, 2001). Imidazole and benzimidazole derivatives are important heteroaromatic compounds and have attracted considerable attention because of their good biological and pharmaceutical activities (Matsuno *et al.*, 2000; Garuti *et al.*, 1999).

In the title compound (Fig. 1), the unsubstituted cyclopentadiene (Cp) ring is disordered over two positions, with site-occupancy factors of 0.636 (12) and 0.364 (12) for the major and minor components respectively. The dihedral angles between the substituted Cp ring and the major and minor components of the disordered ring are 0.8 (3)° and 3.4 (6)°, respectively. All bond lengths and angels are normal. The dihedral angle between the benzimidazole ring system and the substituted Cp ring is 72.92 (9)°. In the crystal structure (Fig. 2), the molecules are connected through intermolecular O—H···O hydrogen bonds (Table 1) to form zigzag chains running parallel to the *a* axis.

### Experimental

The title compound was synthesized by the reaction of L-(-)-1-(1H-benzimidazol-2-yl)ethanol (10 mmol) with a solution of FeCH_2_N^+^(CH_3_)_3_I^-^ (10 mmol) in water (20 ml) at 105 °C. L-(-)-1-(1H-Benzimidazol-2-yl)ethanol was synthesized by the reaction of benzene-1,2-diamine and ethyl L-(-)-lactate at 115°C according to the literature method (Xia & Xu, 2008). Crystals suitable for X-ray diffraction analysis were obtained by slow evaporation of a methanol solution at room temperature over a period of one week.

### Refinement

All H atoms were fixed geometrically and treated as riding, with C—H = 0.93-0.98 Å, O—H = 0.82-0.85 Å and with *U*iso(H) = 1.2*U*eq(C) or 1.5*U*eq(C, O) for methyl, hydroxyl and water H atoms. The C1—C5 cyclopentadiene ring is disordered over two positions, with refined site-occupancy factors of 0.636 (12) and 0.364 (12) for the major and minor components respectively. During the refinement of the disordered cyclopentadiene ring, soft proximity (SIMU) and rigid-bond restraints (DELU) were applied to the anisotropic displacement parameters.

### Crystal data

**Table:**

[Fe(C_5_H_5_)(C_15_H_15_N_2_O)]·H_2_O	*F*(000) = 792
*M**_r_* = 378.25	*D*_x_ = 1.349 Mg m^−^^3^
Orthorhombic, *P*2_1_2_1_2_1_	Mo *K*α radiation, λ = 0.71073 Å
Hall symbol: P 2ac 2ab	Cell parameters from 4425 reflections
*a* = 7.678 (5) Å	θ = 2.7–27.5°
*b* = 12.480 (8) Å	µ = 0.83 mm^−^^1^
*c* = 19.428 (12) Å	*T* = 293 K
*V* = 1862 (2) Å^3^	Block, colourless
*Z* = 4	0.40 × 0.35 × 0.30 mm

### Data collection

**Table:**

Rigaku SCXmini diffractometer	4236 independent reflections
Radiation source: fine-focus sealed tube	3622 reflections with *I* > 2σ(*I*)
graphite	*R*_int_ = 0.042
Detector resolution: 13.6612 pixels mm^-1^	θ_max_ = 27.5°, θ_min_ = 2.7°
ω scans	*h* = −9→9
Absorption correction: multi-scan (*CrystalClear*; Rigaku, 2005)	*k* = −16→16
*T*_min_ = 0.724, *T*_max_ = 0.785	*l* = −25→25
19049 measured reflections	

### Refinement

**Table:**

Refinement on *F*^2^	Secondary atom site location: difference Fourier map
Least-squares matrix: full	Hydrogen site location: inferred from neighbouring sites
*R*[*F*^2^ > 2σ(*F*^2^)] = 0.043	H-atom parameters constrained
*wR*(*F*^2^) = 0.113	*w* = 1/[σ^2^(*F*_o_^2^) + (0.0566*P*)^2^ + 0.1498*P*] where *P* = (*F*_o_^2^ + 2*F*_c_^2^)/3
*S* = 1.05	(Δ/σ)_max_ < 0.001
4236 reflections	Δρ_max_ = 0.32 e Å^−^^3^
243 parameters	Δρ_min_ = −0.21 e Å^−^^3^
146 restraints	Absolute structure: Flack (1983), 1811 Friedel pairs
Primary atom site location: structure-invariant direct methods	Flack parameter: 0.03 (2)

### Special details

**Table:**

Geometry. All e.s.d.'s (except the e.s.d. in the dihedral angle between two l.s. planes) are estimated using the full covariance matrix. The cell e.s.d.'s are taken into account individually in the estimation of e.s.d.'s in distances, angles and torsion angles; correlations between e.s.d.'s in cell parameters are only used when they are defined by crystal symmetry. An approximate (isotropic) treatment of cell e.s.d.'s is used for estimating e.s.d.'s involving l.s. planes.
Refinement. Refinement of *F*^2^ against ALL reflections. The weighted *R*-factor *wR* and goodness of fit *S* are based on *F*^2^, conventional *R*-factors *R* are based on *F*, with *F* set to zero for negative *F*^2^. The threshold expression of *F*^2^ > σ(*F*^2^) is used only for calculating *R*-factors(gt) *etc*. and is not relevant to the choice of reflections for refinement. *R*-factors based on *F*^2^ are statistically about twice as large as those based on *F*, and *R*- factors based on ALL data will be even larger.

### Fractional atomic coordinates and isotropic or equivalent isotropic displacement parameters (Å^2^)

**Table:**

	*x*	*y*	*z*	*U*_iso_*/*U*_eq_	Occ. (<1)
Fe1	0.12377 (5)	0.11965 (3)	0.10136 (2)	0.05548 (14)	
C1	0.2965 (13)	0.2388 (10)	0.0874 (6)	0.076 (2)	0.636 (12)
H1	0.3040	0.3018	0.1128	0.092*	0.636 (12)
C2	0.3868 (13)	0.1411 (10)	0.1064 (6)	0.088 (2)	0.636 (12)
H2	0.4582	0.1309	0.1445	0.105*	0.636 (12)
C3	0.3440 (15)	0.0626 (9)	0.0545 (6)	0.090 (2)	0.636 (12)
H3	0.3799	−0.0085	0.0519	0.108*	0.636 (12)
C4	0.2340 (16)	0.1193 (11)	0.0081 (5)	0.087 (2)	0.636 (12)
H4	0.1888	0.0883	−0.0316	0.104*	0.636 (12)
C5	0.198 (2)	0.2298 (13)	0.0278 (7)	0.081 (2)	0.636 (12)
H5	0.1278	0.2805	0.0063	0.097*	0.636 (12)
C1'	0.344 (2)	0.218 (2)	0.0982 (13)	0.076 (2)	0.364 (12)
H1'	0.3815	0.2628	0.1333	0.092*	0.364 (12)
C2'	0.384 (3)	0.104 (2)	0.0815 (11)	0.088 (2)	0.364 (12)
H2'	0.4599	0.0594	0.1059	0.105*	0.364 (12)
C3'	0.294 (3)	0.0763 (17)	0.0258 (12)	0.090 (2)	0.364 (12)
H3'	0.3024	0.0082	0.0068	0.108*	0.364 (12)
C4'	0.187 (3)	0.1529 (19)	−0.0023 (11)	0.087 (2)	0.364 (12)
H4'	0.1092	0.1521	−0.0391	0.104*	0.364 (12)
C5'	0.236 (4)	0.233 (2)	0.0447 (15)	0.081 (2)	0.364 (12)
H5'	0.1904	0.3009	0.0384	0.097*	0.364 (12)
C6	0.0499 (5)	0.0507 (3)	0.19164 (17)	0.0656 (8)	
H6	0.1226	0.0277	0.2270	0.079*	
C7	−0.0067 (6)	−0.0118 (3)	0.1347 (2)	0.0781 (10)	
H7	0.0243	−0.0825	0.1260	0.094*	
C8	−0.1161 (5)	0.0507 (3)	0.09454 (18)	0.0714 (8)	
H8	−0.1717	0.0286	0.0544	0.086*	
C9	−0.1290 (4)	0.1537 (3)	0.12467 (16)	0.0617 (7)	
H9	−0.1943	0.2108	0.1080	0.074*	
C10	−0.0239 (3)	0.1541 (2)	0.18508 (14)	0.0490 (6)	
C11	−0.0006 (4)	0.2455 (2)	0.23385 (14)	0.0553 (7)	
H11A	0.0989	0.2317	0.2633	0.066*	
H11B	0.0226	0.3105	0.2081	0.066*	
C12	−0.2966 (4)	0.3248 (2)	0.25873 (14)	0.0531 (7)	
C13	−0.3238 (4)	0.3941 (3)	0.20362 (16)	0.0636 (8)	
H13A	−0.2437	0.4009	0.1680	0.076*	
C14	−0.4778 (5)	0.4525 (3)	0.2051 (2)	0.0791 (11)	
H14A	−0.5003	0.5008	0.1698	0.095*	
C15	−0.6005 (6)	0.4410 (4)	0.2581 (2)	0.0918 (12)	
H15A	−0.7020	0.4818	0.2571	0.110*	
C16	−0.5738 (5)	0.3712 (4)	0.3111 (2)	0.0827 (10)	
H16A	−0.6565	0.3628	0.3457	0.099*	
C17	−0.4184 (4)	0.3126 (3)	0.31211 (16)	0.0632 (8)	
C18	−0.1993 (4)	0.2124 (3)	0.33753 (15)	0.0601 (7)	
C19	−0.0719 (5)	0.1412 (3)	0.37575 (19)	0.0757 (10)	
H19A	−0.0051	0.1004	0.3417	0.091*	
C20	−0.1628 (7)	0.0627 (4)	0.4224 (3)	0.1130 (17)	
H20A	−0.0778	0.0186	0.4451	0.169*	
H20B	−0.2393	0.0183	0.3957	0.169*	
H20C	−0.2292	0.1011	0.4563	0.169*	
N1	−0.1568 (3)	0.2606 (2)	0.27640 (11)	0.0538 (6)	
N2	−0.3554 (4)	0.2401 (2)	0.36061 (13)	0.0683 (7)	
O1	0.0467 (4)	0.2076 (3)	0.41300 (18)	0.0971 (9)	
H1''	0.1441	0.1808	0.4120	0.146*	
OW	0.4102 (5)	0.1890 (3)	0.47061 (15)	0.1251 (12)	
HWB	0.4440	0.2535	0.4746	0.188*	
HWC	0.3948	0.1621	0.5104	0.188*	

### Atomic displacement parameters (Å^2^)

**Table:**

	*U*^11^	*U*^22^	*U*^33^	*U*^12^	*U*^13^	*U*^23^
Fe1	0.0499 (2)	0.0611 (2)	0.0554 (2)	0.0041 (2)	0.01120 (19)	0.0056 (2)
C1	0.042 (5)	0.101 (5)	0.086 (5)	−0.012 (3)	0.007 (4)	0.018 (4)
C2	0.051 (2)	0.129 (7)	0.084 (6)	0.007 (4)	0.017 (4)	0.021 (4)
C3	0.072 (6)	0.119 (4)	0.079 (6)	0.029 (3)	0.028 (4)	0.005 (4)
C4	0.079 (7)	0.124 (7)	0.058 (4)	0.009 (4)	0.019 (3)	0.016 (4)
C5	0.068 (7)	0.104 (3)	0.071 (7)	−0.004 (4)	0.012 (3)	0.029 (4)
C1'	0.042 (5)	0.101 (5)	0.086 (5)	−0.012 (3)	0.007 (4)	0.018 (4)
C2'	0.051 (2)	0.129 (7)	0.084 (6)	0.007 (4)	0.017 (4)	0.021 (4)
C3'	0.072 (6)	0.119 (4)	0.079 (6)	0.029 (3)	0.028 (4)	0.005 (4)
C4'	0.079 (7)	0.124 (7)	0.058 (4)	0.009 (4)	0.019 (3)	0.016 (4)
C5'	0.068 (7)	0.104 (3)	0.071 (7)	−0.004 (4)	0.012 (3)	0.029 (4)
C6	0.0729 (19)	0.0610 (17)	0.0627 (18)	0.0124 (15)	0.0137 (16)	0.0162 (15)
C7	0.099 (3)	0.0551 (18)	0.081 (2)	−0.0086 (19)	0.025 (2)	−0.0063 (17)
C8	0.0674 (19)	0.083 (2)	0.0638 (18)	−0.0155 (18)	−0.002 (2)	−0.0108 (17)
C9	0.0454 (14)	0.083 (2)	0.0564 (15)	0.0054 (15)	−0.0020 (13)	−0.0046 (14)
C10	0.0421 (13)	0.0572 (15)	0.0476 (13)	0.0021 (11)	0.0044 (11)	0.0056 (12)
C11	0.0479 (14)	0.0623 (17)	0.0559 (17)	0.0006 (13)	−0.0042 (12)	0.0015 (14)
C12	0.0512 (15)	0.0579 (16)	0.0501 (15)	0.0052 (13)	−0.0090 (12)	−0.0099 (12)
C13	0.0656 (18)	0.0669 (19)	0.0582 (16)	0.0019 (15)	−0.0125 (14)	−0.0036 (15)
C14	0.080 (2)	0.075 (2)	0.082 (2)	0.0162 (19)	−0.033 (2)	−0.0011 (19)
C15	0.077 (3)	0.099 (3)	0.099 (3)	0.031 (2)	−0.018 (2)	−0.020 (2)
C16	0.066 (2)	0.101 (3)	0.081 (2)	0.021 (2)	0.0003 (18)	−0.021 (2)
C17	0.0593 (18)	0.0718 (19)	0.0586 (17)	0.0068 (14)	−0.0012 (14)	−0.0115 (16)
C18	0.071 (2)	0.0627 (18)	0.0471 (14)	0.0018 (15)	−0.0013 (14)	−0.0036 (13)
C19	0.084 (2)	0.077 (2)	0.0655 (18)	0.0096 (18)	−0.0145 (18)	0.0093 (17)
C20	0.129 (4)	0.099 (3)	0.111 (3)	−0.020 (3)	−0.046 (3)	0.045 (3)
N1	0.0567 (14)	0.0597 (13)	0.0449 (11)	0.0061 (11)	−0.0005 (11)	−0.0005 (10)
N2	0.0730 (17)	0.0795 (17)	0.0523 (13)	0.0063 (15)	0.0105 (14)	−0.0061 (12)
O1	0.0643 (15)	0.121 (2)	0.106 (2)	−0.0134 (15)	−0.0203 (16)	0.0323 (18)
OW	0.124 (3)	0.177 (3)	0.0739 (17)	0.012 (3)	0.0232 (18)	0.0031 (19)

### Geometric parameters (Å, °)

**Table:**

Fe1—C5'	1.98 (3)	C7—C8	1.387 (5)
Fe1—C4	2.000 (10)	C7—H7	0.9300
Fe1—C1	2.011 (12)	C8—C9	1.416 (5)
Fe1—C7	2.029 (4)	C8—H8	0.9300
Fe1—C10	2.029 (3)	C9—C10	1.424 (4)
Fe1—C6	2.034 (3)	C9—H9	0.9300
Fe1—C8	2.037 (4)	C10—C11	1.494 (4)
Fe1—C9	2.038 (3)	C11—N1	1.469 (4)
Fe1—C3'	2.036 (17)	C11—H11A	0.9700
Fe1—C2	2.040 (10)	C11—H11B	0.9700
Fe1—C2'	2.05 (2)	C12—N1	1.383 (4)
Fe1—C3	2.048 (8)	C12—C13	1.392 (4)
C1—C5	1.388 (12)	C12—C17	1.404 (4)
C1—C2	1.451 (13)	C13—C14	1.390 (5)
C1—H1	0.9300	C13—H13A	0.9300
C2—C3	1.444 (14)	C14—C15	1.403 (6)
C2—H2	0.9300	C14—H14A	0.9300
C3—C4	1.423 (10)	C15—C16	1.365 (6)
C3—H3	0.9300	C15—H15A	0.9300
C4—C5	1.46 (2)	C16—C17	1.400 (5)
C4—H4	0.9300	C16—H16A	0.9300
C5—H5	0.9300	C17—N2	1.393 (4)
C1'—C5'	1.34 (2)	C18—N2	1.325 (4)
C1'—C2'	1.49 (3)	C18—N1	1.370 (4)
C1'—H1'	0.9300	C18—C19	1.516 (5)
C2'—C3'	1.33 (2)	C19—O1	1.428 (5)
C2'—H2'	0.9300	C19—C20	1.507 (6)
C3'—C4'	1.37 (2)	C19—H19A	0.9800
C3'—H3'	0.9300	C20—H20A	0.9600
C4'—C5'	1.40 (4)	C20—H20B	0.9600
C4'—H4'	0.9300	C20—H20C	0.9600
C5'—H5'	0.9300	O1—H1''	0.8200
C6—C10	1.415 (4)	OW—HWB	0.8501
C6—C7	1.421 (5)	OW—HWC	0.8500
C6—H6	0.9300		
			
C5'—Fe1—C4	46.8 (9)	Fe1—C1'—H1'	125.5
C5'—Fe1—C1	27.6 (8)	C3'—C2'—C1'	108.2 (18)
C4—Fe1—C1	66.5 (5)	C3'—C2'—Fe1	70.5 (12)
C5'—Fe1—C7	165.0 (8)	C1'—C2'—Fe1	70.4 (12)
C4—Fe1—C7	119.8 (4)	C3'—C2'—H2'	125.9
C1—Fe1—C7	165.2 (3)	C1'—C2'—H2'	125.9
C5'—Fe1—C10	122.4 (9)	Fe1—C2'—H2'	124.8
C4—Fe1—C10	164.4 (4)	C2'—C3'—C4'	117 (2)
C1—Fe1—C10	108.7 (4)	C2'—C3'—Fe1	71.4 (11)
C7—Fe1—C10	68.85 (13)	C4'—C3'—Fe1	73.6 (13)
C5'—Fe1—C6	154.1 (8)	C2'—C3'—H3'	121.4
C4—Fe1—C6	153.9 (4)	C4'—C3'—H3'	121.4
C1—Fe1—C6	127.8 (4)	Fe1—C3'—H3'	125.2
C7—Fe1—C6	40.95 (14)	C3'—C4'—C5'	94.2 (18)
C10—Fe1—C6	40.76 (12)	C3'—C4'—Fe1	67.7 (12)
C5'—Fe1—C8	131.0 (7)	C5'—C4'—Fe1	65.2 (16)
C4—Fe1—C8	108.8 (4)	C3'—C4'—H4'	132.9
C1—Fe1—C8	154.1 (3)	C5'—C4'—H4'	132.9
C7—Fe1—C8	39.88 (15)	Fe1—C4'—H4'	125.9
C10—Fe1—C8	68.68 (13)	C1'—C5'—C4'	125 (2)
C6—Fe1—C8	68.00 (15)	C1'—C5'—Fe1	75.1 (16)
C5'—Fe1—C9	112.9 (8)	C4'—C5'—Fe1	74.9 (17)
C4—Fe1—C9	127.2 (4)	C1'—C5'—H5'	117.5
C1—Fe1—C9	120.3 (3)	C4'—C5'—H5'	117.5
C7—Fe1—C9	68.11 (16)	Fe1—C5'—H5'	124.1
C10—Fe1—C9	41.00 (11)	C10—C6—C7	107.9 (3)
C6—Fe1—C9	68.35 (13)	C10—C6—Fe1	69.42 (17)
C8—Fe1—C9	40.68 (14)	C7—C6—Fe1	69.3 (2)
C5'—Fe1—C3'	60.7 (11)	C10—C6—H6	126.0
C4—Fe1—C3'	22.4 (5)	C7—C6—H6	126.0
C1—Fe1—C3'	71.2 (7)	Fe1—C6—H6	126.8
C7—Fe1—C3'	109.4 (6)	C8—C7—C6	108.3 (3)
C10—Fe1—C3'	172.7 (8)	C8—C7—Fe1	70.4 (2)
C6—Fe1—C3'	133.4 (7)	C6—C7—Fe1	69.73 (19)
C8—Fe1—C3'	114.8 (6)	C8—C7—H7	125.8
C9—Fe1—C3'	145.6 (7)	C6—C7—H7	125.8
C5'—Fe1—C2	60.2 (7)	Fe1—C7—H7	125.6
C4—Fe1—C2	68.0 (4)	C7—C8—C9	108.7 (3)
C1—Fe1—C2	42.0 (3)	C7—C8—Fe1	69.7 (2)
C7—Fe1—C2	125.4 (3)	C9—C8—Fe1	69.69 (19)
C10—Fe1—C2	119.1 (4)	C7—C8—H8	125.7
C6—Fe1—C2	106.9 (3)	C9—C8—H8	125.7
C8—Fe1—C2	162.5 (4)	Fe1—C8—H8	126.5
C9—Fe1—C2	154.8 (4)	C8—C9—C10	107.7 (3)
C3'—Fe1—C2	55.6 (6)	C8—C9—Fe1	69.6 (2)
C5'—Fe1—C2'	62.6 (10)	C10—C9—Fe1	69.17 (17)
C4—Fe1—C2'	54.3 (6)	C8—C9—H9	126.1
C1—Fe1—C2'	53.3 (7)	C10—C9—H9	126.1
C7—Fe1—C2'	117.7 (7)	Fe1—C9—H9	126.6
C10—Fe1—C2'	135.9 (7)	C6—C10—C9	107.3 (3)
C6—Fe1—C2'	113.2 (6)	C6—C10—C11	126.2 (3)
C8—Fe1—C2'	146.1 (8)	C9—C10—C11	126.4 (3)
C9—Fe1—C2'	173.2 (8)	C6—C10—Fe1	69.82 (17)
C3'—Fe1—C2'	38.1 (7)	C9—C10—Fe1	69.83 (17)
C2—Fe1—C2'	18.9 (6)	C11—C10—Fe1	127.10 (19)
C5'—Fe1—C3	69.0 (9)	N1—C11—C10	110.9 (2)
C4—Fe1—C3	41.1 (3)	N1—C11—H11A	109.5
C1—Fe1—C3	69.7 (5)	C10—C11—H11A	109.5
C7—Fe1—C3	105.6 (3)	N1—C11—H11B	109.5
C10—Fe1—C3	153.1 (4)	C10—C11—H11B	109.5
C6—Fe1—C3	117.8 (4)	H11A—C11—H11B	108.1
C8—Fe1—C3	124.8 (4)	N1—C12—C13	131.8 (3)
C9—Fe1—C3	163.2 (4)	N1—C12—C17	105.7 (3)
C3'—Fe1—C3	19.7 (5)	C13—C12—C17	122.4 (3)
C2—Fe1—C3	41.4 (4)	C14—C13—C12	115.9 (3)
C2'—Fe1—C3	22.5 (5)	C14—C13—H13A	122.0
C5—C1—C2	113.8 (11)	C12—C13—H13A	122.0
C5—C1—Fe1	72.1 (9)	C13—C14—C15	122.2 (4)
C2—C1—Fe1	70.1 (6)	C13—C14—H14A	118.9
C5—C1—H1	123.1	C15—C14—H14A	118.9
C2—C1—H1	123.1	C16—C15—C14	121.2 (4)
Fe1—C1—H1	126.5	C16—C15—H15A	119.4
C3—C2—C1	106.5 (8)	C14—C15—H15A	119.4
C3—C2—Fe1	69.6 (5)	C15—C16—C17	118.2 (4)
C1—C2—Fe1	68.0 (6)	C15—C16—H16A	120.9
C3—C2—H2	126.8	C17—C16—H16A	120.9
C1—C2—H2	126.8	N2—C17—C16	130.1 (3)
Fe1—C2—H2	127.2	N2—C17—C12	109.8 (3)
C4—C3—C2	103.9 (8)	C16—C17—C12	120.1 (3)
C4—C3—Fe1	67.6 (5)	N2—C18—N1	113.2 (3)
C2—C3—Fe1	69.0 (5)	N2—C18—C19	124.8 (3)
C4—C3—H3	128.0	N1—C18—C19	121.9 (3)
C2—C3—H3	128.0	O1—C19—C20	111.6 (3)
Fe1—C3—H3	126.9	O1—C19—C18	108.6 (3)
C3—C4—C5	114.6 (10)	C20—C19—C18	112.2 (4)
C3—C4—Fe1	71.2 (5)	O1—C19—H19A	108.1
C5—C4—Fe1	71.3 (8)	C20—C19—H19A	108.1
C3—C4—H4	122.7	C18—C19—H19A	108.1
C5—C4—H4	122.7	C19—C20—H20A	109.5
Fe1—C4—H4	126.5	C19—C20—H20B	109.5
C1—C5—C4	101.1 (10)	H20A—C20—H20B	109.5
C1—C5—Fe1	68.1 (8)	C19—C20—H20C	109.5
C4—C5—Fe1	66.7 (8)	H20A—C20—H20C	109.5
C1—C5—H5	129.4	H20B—C20—H20C	109.5
C4—C5—H5	129.4	C18—N1—C12	106.5 (2)
Fe1—C5—H5	127.2	C18—N1—C11	128.8 (3)
C5'—C1'—C2'	95 (2)	C12—N1—C11	124.6 (2)
C5'—C1'—Fe1	66.6 (17)	C18—N2—C17	104.7 (3)
C2'—C1'—Fe1	67.4 (12)	C19—O1—H1''	109.5
C5'—C1'—H1'	132.3	HWB—OW—HWC	109.5
C2'—C1'—H1'	132.3		

### Hydrogen-bond geometry (Å, °)

**Table:**

*D*—H···*A*	*D*—H	H···*A*	*D*···*A*	*D*—H···*A*
OW—HWB···O1^i^	0.85	2.37	2.806 (5)	112
O1—H1''···OW	0.82	2.34	3.016 (5)	140

Symmetry codes: (i) *x*+1/2, −*y*+1/2, −*z*+1.
